# Time-Rescaling of Dirac Dynamics: Shortcuts to Adiabaticity in Ion Traps and Weyl Semimetals

**DOI:** 10.3390/e23010081

**Published:** 2021-01-08

**Authors:** Agniva Roychowdhury, Sebastian Deffner

**Affiliations:** 1 Department of Physics, University of Maryland, Baltimore County, Baltimore, MD 21250, USA; deffner@umbc.edu; 2 Instituto de Física ‘Gleb Wataghin’, Universidade Estadual de Campinas, Campinas 13083-859, São Paulo, Brazil

**Keywords:** shortcuts to adiabaticity, quantum control, Dirac dynamics, ion traps, Weyl semimetals

## Abstract

Only very recently, rescaling time has been recognized as a way to achieve adiabatic dynamics in fast processes. The advantage of time-rescaling over other shortcuts to adiabaticity is that it does not depend on the eigenspectrum and eigenstates of the Hamiltonian. However, time-rescaling requires that the original dynamics are adiabatic, and in the rescaled time frame, the Hamiltonian exhibits non-trivial time-dependence. In this work, we show how time-rescaling can be applied to Dirac dynamics, and we show that all time-dependence can be absorbed into the effective potentials through a judiciously chosen unitary transformation. This is demonstrated for two experimentally relevant scenarios, namely for ion traps and adiabatic creation of Weyl points.

## 1. Introduction

From the very beginning of quantum control, it has been recognized that circumventing the quantum adiabatic theorem [1] poses a formidable challenge. In essence, this theorem asserts that any quantum process that is driven at rates larger than the typical energy gaps, is inevitably accompanied by excitations [2,3,4,5]. In the real world, these parasitic excitations are often not only undesirable, but even detrimental. For instance, in adiabatic quantum computing [6], finite-time effects constitute a major source for computational errors [7,8]. Thus, to circumvent, mitigate, and suppress such finite-time excitations in controlled quantum processes, a wide variety of techniques has been developed. Among the most successful approaches are transitionless quantum driving [9,10,11,12,13], the fast-forward technique [14,15,16,17], and methods that rely on identifying the adiabatic invariants [18,19,20,21], to name just a few. For a comprehensive exposition of the field “shortcuts to adiabaticity”, we refer to recent reviews [22,23], a special collection of articles [24], and a perspective [25].

Somewhat naturally, the majority of work has focused on quantum processes that can be described by time-dependent Schrödinger equations. However, shortcuts to adiabaticty have also found generalizations and applications in, e.g., open system dynamics [26], classical dynamics [27,28], and even biologically relevant settings [29]. Complementing these efforts, the present paper focuses on relativistic quantum dynamics. This is motivated by recent work that has generalized the fast-forward technique [30], transitionless quantum driving [31], and invariant based methods [32] to controlled Dirac dynamics.

The Dirac equation [33] was originally formulated to describe the properties of massive spin-1/2 particles, such as electrons and quarks [34,35]. However, in recent years, it has attracted wider attention [36,37,38,39,40,41,42,43], which is mostly motivated by the discovery of so-called Dirac materials [44]. In these systems, the dispersion relation becomes linear, and hence the low-energy excitations behave more akin to massless Dirac particles than fermionic Schrödinger particles.

In the context of shortcuts to adiabaticity, the technical challenges already present for Schrödinger dynamics become significantly more involved for Dirac dynamics [45]. One way or another, implementing most shortcuts requires knowledge of the energy spectrum, or the use of highly non-local control fields. Therefore, any technique that requires less detailed information about the dynamics appears highly desirable [25]. In the following, we propose and demonstrate how the method of “time-rescaling” [46] is generalized to Dirac dynamics. We will see that, while simply applying a scaling transformation is mathematically straight forward, the potential physical implementations are markedly less clear. This originates in the fact that rescaling time leads to an effectively time-dependent mass [46]. We will show how this can be remedied by a judiciously chosen unitary transformation of the Dirac equation. The experimental applicability of our findings is demonstrated for two relevant systems, namely for ion traps and adiabatic pumping in Weyl semimetals.

Due to the wide variety of concepts used in the following analysis, the narrative has been written as self-contained as possible. In Section 2, we summarize the main properties of the Dirac equation, and briefly review time-rescaling for Schrödinger dynamics. In Section 3, we develop the method of time-rescaling for general Dirac dynamics. Section 4 is dedicated to adiabatically driving laser ion traps, and Section 5 presents adiabatic pumping in Weyl semimetals. Finally, the analysis is concluded with a few remarks in Section 6.

## 2. Preliminaries

We start by outlining notions and notations, and by briefly reviewing instrumental results from the literature.

### 2.1. Relativistic Quantum Mechanics: The Dirac Equation

The Dirac equation has its origin in an attempt to reconcile special relativity and quantum mechanics [33]. In its original inception and in first quantization, it correctly describes the properties of massive spin-1/2 particles. It can be written in space representation as [34],

(1) $$i\hbar \;\dot{\Psi }\left(x,t\right)=\left[\alpha \cdot \left(-i\hbar c\;\nabla +A(x,t)\right)+\alpha_{0}\;mc^{2}+I_{4}\;V\left(x,t\right)\right]\;\Psi \left(x,t\right)\;.$$

Here, Ψ(x,t), the 4-dimensional Dirac spinor, i.e., the wave function of a charged spin-1/2 particle with rest mass *m* at position $x=(x_{1},x_{2},x_{3})$, and *c* is the speed of light. As usual, we denote the derivative with respect to time by a dot.

In covariant form the matrices $\alpha =(\alpha_{1},\alpha_{2},\alpha_{3})$ and $\alpha_{0}$ can be expressed as [34,35],

(2) $$\alpha^{0}=\gamma^{0}\;\mathrm{and}\;\gamma^{0}\;\alpha^{k}=\gamma^{k}\;.$$

The γ-matrices are commonly written in terms of 2×2 sub-matrices with the Pauli-matrices $\sigma_{x},\sigma_{y},\sigma_{z}$ and the identity $I_{2}$ as,

(3) $$\begin{array}{ccc} \gamma^{0}=\left(\begin{array}{cc} I_{2} & 0\\ 0 & -I_{2} \end{array}\right)\; & \gamma^{1}=\left(\begin{array}{cc} 0 & \sigma_{x}\\ -\sigma_{x} & 0 \end{array}\right)\;\gamma^{2}=\left(\begin{array}{cc} 0 & \sigma_{y}\\ -\sigma_{y} & 0 \end{array}\right)\; & \gamma^{3}=\left(\begin{array}{cc} 0 & \sigma_{z}\\ -\sigma_{z} & 0 \end{array}\right)\;. \end{array}$$

Finally, A(x,t) is the vector potential, and V(x,t) is the scalar potential. The electric and magnetic fields, E(x,t) and B(x,t), are given by

(4) $$E\left(x,t\right)=-\nabla \;V\left(x,t\right)-\dot{A}\left(x,t\right)\;\mathrm{and}\;B\left(x,t\right)=\nabla \times A\left(x,t\right)\;.$$

Note that the Dirac equation is gauge invariant [35], and we can thus choose mathematically convenient representations.

In the following, it will also prove convenient to introduce the Dirac–Hamiltonian $H_{D}$, with which Equation (1) can be expressed in basis-independent form,

(5) $$i\hbar \;|\dot{\Psi }\left(t\right)\rangle =H_{D}\left(t\right)\;|\Psi (t)\rangle \;.$$

Hence, the Dirac Equation (5) becomes formally identical to the time-dependent Schrödinger equation. It is worth emphasizing, however, that the standard Schrödinger Hamiltonian is quadratic in momentum, whereas the Dirac–Hamiltonian is linear. This is a direct consequence of the relativistic energy–momentum relation, and it will become instrumental in the following analysis. Moreover, for massive particles, the Dirac–Hamiltonian contains the rest energy, which is not present in pure spin systems or in Schrödinger dynamics. We will see in the following that when rescaling time, this additional term requires special attention.

### 2.2. Time-Rescaling of Schrödinger Dynamics

Time-rescaling was put forward by Bernardo in Ref. [46] as an alternative method for finding shortcuts to adiabaticity, that does not depend on the instantaneous eigenstates of the dynamics. To this end, Ref. [46] considers the time-dependent Schrödinger equation

(6) $$i\hbar \;|\dot{\psi }\left(t\right)\rangle =\left(\frac{p^{2}}{2m}+V\left(x,t\right)\right)\;|\psi (t)\rangle =H\left(t\right)\;|\psi (t)\rangle \;,$$

where H(t) is the standard Hamiltonian. The solution of Equation (6) can be expressed in terms of the unitary evolution operator,

(7) $$U\left(\tau \right)=T_{>}\;\exp\left\{-\frac{i}{\hbar }\int_{0}^{\tau }dt\;H\left(t\right)\right\}$$

where $T_{>}$ denotes time-ordering.

Time-rescaling is then nothing else but a transformation of the time variable in the exponent of the unitary evolution. We have

(8) $$U\left(\tau \right)=T_{>}\;\exp\left\{-\frac{i}{\hbar }\int_{f^{-1}\left(0\right)}^{f^{-1}\left(\tau \right)}ds\;\dot{f}\left(s\right)\;H\left(f\left(s\right)\right)\right\}\;,$$

where f(t) is an arbitrary rescaling function. It is then easy to see that Equation (8) can be exploited as a shortcut to adiabaticity for any $\left|f^{-1}\left(\tau \right)-f^{-1}\left(0\right)\right|\leq \tau$. If the original dynamics (7) describes an adiabatic process, then Equation (8) achieves the same adiabatic dynamics in shorter time, for all rescaling functions f(t) that obey the boundary conditions

(9) $$f^{-1}\left(0\right)=0,\;f^{-1}\left(\tau \right)=\tau /a,\;\mathrm{and}\;\dot{f}\left(0\right)=\dot{f}\left(\tau /a\right)=1$$

where a>1 determines the “time contraction factor” [46].

The shortcoming of this technique is that in rescaled variables, the new Hamiltonian becomes $\tilde{H}\left(t\right)\equiv \dot{f}\left(t\right)H\left(f\left(t\right)\right)$. Generically, this leads to a time-dependent mass, which is not easy to realize in experimental scenarios. In Appendix A, we show how this can be remedied with the help of a canonical transformation. In particular, we show that time-rescaling and counterdiabetic driving are equivalent for scale-invariant problems [13].

In the following, we will generalize and analyze Equation (8) for the time-dependent Dirac Equation (5). Special focus will be put on experimental accessibility of the arising time-dependent terms.

## 3. Time-Rescaling of Dirac Dynamics

It is easy to see that the solution of the time-dependent Dirac Equation (5) can be expressed as

(10) $$U_{D}\left(\tau \right)=T_{>}\;\exp\left\{-\frac{i}{\hbar }\int_{0}^{\tau }dt\;H_{D}\left(t\right)\right\}\;.$$

Hence, the time-rescaled dynamics become

(11) $$U_{D}\left(\tau \right)=T_{>}\;\exp\left\{-\frac{i}{\hbar }\int_{f^{-1}\left(0\right)}^{f^{-1}\left(\tau \right)}ds\;{\tilde{H}}_{D}\left(s\right)\right\}\;,$$

where ${\tilde{H}}_{D}\left(t\right)=\dot{f}\left(t\right)\;H_{D}\left(f\left(t\right)\right)$ is the time-rescaled Dirac–Hamiltonian.

Hence, it appears to be rather straightforward to employ time-rescaling as a shortcut to adiabaticity also in Dirac dynamics. That the situation is not quite that simple becomes apparent when inspecting the explicit form of the Dirac–Hamiltonian (1). Notice, that while time-rescaling Schrödinger dynamics only led to a time-dependent mass, for Dirac dynamics, the time-scaled Hamiltonian ${\tilde{H}}_{D}\left(t\right)$ is governed by an effectively time-dependent speed of light, $\tilde{c}\left(t\right)=\dot{f}\left(t\right)\;c$, and an effectively time-dependent rest energy $\tilde{m}\left(t\right)\tilde{c}{\left(t\right)}^{2}=\dot{f}\left(t\right)\;mc^{2}$.

We will see in the following Section 4 that considering $\tilde{c}\left(t\right)$ and $\tilde{m}\left(t\right)$ is perfectly reasonable and realizable in ion traps, which are described by an effective Dirac equation. In general, however, it seems rather implausible that the speed of light and the rest energy can be considered time-dependent control parameters. Thus, we continue the analysis by proposing a canonical transformation that maps the effective time dependence exclusively onto vector and scalar potential, A(x,t) and V(x,t).

For the sake of simplicity, and without loss of generality, we continue by considering a system that is restricted to the *x*-direction. In this case, the 4-component Dirac spinor can be separated into two identical 2-component bispinors. For mathematical convenience, we choose a representation in which the (1+1)-dimensional Dirac equation reads [47,48],

(12) $${\tilde{H}}_{D}\left(t\right)=\left[\tilde{c}\left(t\right)\;p+\tilde{A}\left(x,t\right)\right]\sigma_{x}+\tilde{m}\left(t\right)\tilde{c}{\left(t\right)}^{2}\;\sigma_{z}+\tilde{V}\left(x,t\right)\;I_{2}\;,$$

and where we introduced the time-rescaled potentials $\tilde{A}\left(x,t\right)=\dot{f}\left(t\right)A\left(x,t\right)$ and $\tilde{V}\left(x,t\right)=\dot{f}\left(t\right)V\left(x,t\right)$.

### Absorbing the Time-Dependence into the Potentials

Our goal is now to find a unitary transformation that allows one to write the time-rescaled Hamiltonian in standard form, i.e., with time-independent speed of light and rest mass. Arguably, the simplest ansatz is given by

(13) $$K\left(t\right)=\exp\left\{i\phi \left(t\right)\;\sigma_{x}\right\}=\cos\left(\phi \left(t\right)\right)\;I_{2}+i\sin\left(\phi \left(t\right)\right)\;\sigma_{x}\;.$$

Thus, we obtain

(14) $$\begin{array}{cc} H_{D}\left(t\right) & =K^{†}\left(t\right)\;{\tilde{H}}_{D}\left(x,p,t\right)\;K\left(t\right)-i\hbar \;K^{†}\left(t\right)\;\partial_{t}\;K\left(t\right)\\ \; & =\left[\tilde{c}\left(t\right)\;p+\tilde{A}\left(x,t\right)+\dot{\phi }\left(t\right)\right]\;\sigma_{x}+\tilde{m}\left(t\right)\tilde{c}{\left(t\right)}^{2}\;\cos\left(2\phi \left(t\right)\right)\;\sigma_{z}\\ \; & \;+\tilde{m}\left(t\right)\tilde{c}{\left(t\right)}^{2}\;\sin\left(2\phi \left(t\right)\right)\;\sigma_{y}+\tilde{V}\left(x,t\right)\;I_{2}\;, \end{array}$$

and the corresponding solution is given by Φ(x,t)=K(t)Ψ(x,t). We immediately observe that the time-dependent rest energy is multiplied by cos(2ϕ(t)), whereas the kinetic term is modified by $\dot{\phi }\left(t\right)$. Therefore, the two effectively time-dependent quantities, m(t) and c(t), can be fully described by ϕ(t) and its derivative $\dot{\phi }\left(t\right)$, respectively.

We start by choosing

(15) $$\cos\left(2\phi \left(t\right)\right)=1/\dot{f}\left(t\right)$$

for which the rest energy becomes time-independent. In complete analogy to the Schrödinger case [46], the time-dependence of the kinetic term can then be absorbed into the vector potential. In particular, we define

(16) $$A\left(x,p,t\right)\equiv \tilde{A}\left(x,t\right)+\left(\dot{f}\left(t\right)-1\right)\;c\;p+\frac{\ddot{f}\left(t\right)}{2\dot{f}\left(t\right)\;\sqrt{{\left[\dot{f}\left(t\right)\right]}^{2}-1}}\;,$$

which is nothing else but $\tilde{A}\left(x,t\right)$ expressed in the corresponding interaction picture, plus a position-independent term.

Thus, we are only left with the pseudoscalar term [49,50], that is proportional to $\sigma_{y}$. Pseudoscalar potentials correspond physically to driving the system with circularly polarized light. Now, defining a new (scalar) potential

(17) $$V\left(x,t\right)\equiv mc^{2}\;\sqrt{{\left[\dot{f}\left(t\right)\right]}^{2}-1}\;\sigma_{y}+\tilde{V}\left(x,t\right)\;I_{2}\;,$$

we finally obtain

(18) $$H_{D}\left(t\right)=\left[cp+A(x,p,t)\right]\;\sigma_{x}+mc^{2}\;\sigma_{z}+V\left(x,t\right)\;,$$

where all the time dependence has been absorbed into the potentials. At first glance, the momentum-dependent vector potential may look unphysical. However, this is simply a consequence of the fact that the system is non-conservative, and hence the driven system will experience an intertial force [51], due to the “acceleration” from the time-rescaling.

In conclusion, we have shown that, while in Dirac dynamics we have two, instead of only one, effectively time-dependent parameters, a simple unitary transformation allows to absorb the time-dependence entirely into vector and scalar potentials. The resulting vector potential is fully analogous to what was proposed in Ref. [46] for Schrödinger dynamics, and the scalar potential contains a simple pseudoscalar term.

## 4. Dirac Dynamics in Laser Ion-Traps

It has been experimentally demonstrated that under certain conditions ions in laser traps can be described by effective Dirac dynamics [52,53,54,55]. In general, the applied laser field couples internal vibrational levels of the ion and its motional degrees of freedom. Hence, the total Hamiltonian reads,

(19) $$H_{\mathrm{tot}}=H_{m}+H_{e}+H_{\mathrm{int}}$$

where $H_{m}$ and $H_{e}$ describe motional and electronic degrees of freedom, respectively.

The interaction Hamiltonian $H_{\mathrm{int}}$ can be written in (1 + 1) dimensions as [53]

(20) $$H_{\mathrm{int}}\left(t\right)=2\eta \;\Delta \;\gamma \;\left[p-A\left(t\right)\right]\;\sigma_{x}+\hbar \omega \;\sigma_{z}$$

where $\eta =k\sqrt{\hbar /2m\nu }$ is the Lamb–Dicke parameter, *m* denotes the mass of the trapped ion, and ν is the axial frequency of the confining Paul trap. Note that the effective interaction can be tuned by applying an external magnetic field described by A(t). Furthermore, $\Delta =\sqrt{\hbar /2m\nu }$ and is the width the ground-state wave-function, and γ is the strength of the interaction, which is varied by modulating the laser source. Finally, ω describes the detuning of the laser and the resonance frequency of the two-level atom. In principle, both γ and ω can be controlled externally, and varied as a function of time.

We immediately recognize $H_{\mathrm{int}}$ as the Dirac–Hamiltonian (12) for a vanishing scalar potential, and [53]

(21) $$\tilde{c}\left(t\right)=2\eta \;\Delta \;\gamma \left(t\right)\;\mathrm{and}\;\tilde{m}\left(t\right)\tilde{c}{\left(t\right)}^{2}=\hbar \omega \left(t\right)\;.$$

Hence, $H_{\mathrm{int}}$ is already in the time-dependent form required to implement a shortcut to adiabaticity by means of time-rescaling.

### A Simple Demonstration

Before we continue, it is instructive to demonstrate time-rescaled Dirac dynamics in ion traps with a simple example. To this end, consider a simple scenario, in which we again choose V(x,t)=0. In the interaction picture, we have

(22) $$H_{D}\left(p,t\right)=H_{\mathrm{int}}\left(t\right)=\left[p-\sin^{2}\left(\pi t/2\tau \right)\right]\;\sigma_{x}+\cos^{2}\left(\pi t/2\tau \right)\;\sigma_{z}.$$

Note that the latter Hamiltonian is written in instantaneous units of 2ηΔγ(t). Thus, we simply have $A\left(t\right)=\sin^{2}\left(\pi t/2\tau \right)$ and $\hbar \omega \left(t\right)=\cos^{2}\left(\pi t/2\tau \right)$. Its instantaneous eigenstates can be expressed as

(23) $$|\Phi (t)\rangle =\cos\left(\theta_{t}/2\right)|0\rangle +\sin\left(\theta_{t}/2\right)|1\rangle$$

where $|0\rangle$ and $|1\rangle$ are basis states of $\sigma_{z}$ and $\theta_{t}=\mathrm{Arctan}\left[\left(p_{t}-A\left(t\right)\right)/\hbar \omega \left(t\right)\right]$, where $p_{t}$ is the instantaneous momentum. We immediately observe that for large enough τ, this simple Dirac–Hamiltonian (22) describes adiabatic dynamics, i.e., a system prepared in an initial eigenstate will remain in the corresponding, instantaneous eigenstate.

Now let us assume that the desired target state of the dynamics is $|\Phi_{\mathrm{target}}\rangle =\left(1/\sqrt{2}\right)\left(|0\rangle +|1\rangle \right)$, which is the instantaneous eigenstate of $H_{D}\left(p,t\right)$ at t=τ. Using time-rescaling, this target state can be reached in a shorter time. To this end, we choose the rescaling function f(t) [46] to read

(24) $$f\left(t\right)=a\;t-\frac{\tau \;(a-1)}{2\pi a}\;\sin\left(\frac{2\pi at}{\tau }\right)$$

where as before, a≥1 is the acceleration factor. It is easy to see [46] that this choice fulfills all boundary conditions (9), and notice that, for a=1, we simply have f(t)=t, i.e., we recover the original dynamics.

It is then a simple exercise to solve the dynamics numerically for the original Hamiltonian, $H_{D}\left(t\right)$, (22) and its time-rescaled companion, ${\tilde{H}}_{D}\left(t\right)=\dot{f}\left(t\right)\;H_{D}\left(t\right)$. To illustrate our findings, we choose the system to be initially prepared in $|\Phi (0)\rangle$ (23), and compute the fidelity of the time-evolved state with respect to initial and target state, $F_{i}\left(t\right)$ and $F_{f}\left(t\right)$, respectively. These are given by

(25) $$F_{i}\left(t\right)={\left|\int_{-\infty }^{+\infty }dp\;\langle \Psi (p,t)|\Phi (0)\rangle \right|}^{2}\;\mathrm{and}\;F_{f}\left(t\right)={\left|\int_{-\infty }^{+\infty }dp\;\langle \Psi \left(p,t\right)|\Phi_{\mathrm{target}}\rangle \right|}^{2}\;.$$

**Figure 1:**
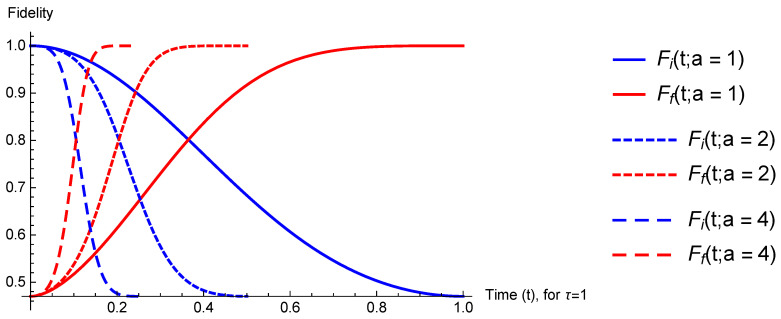
Fidelity of the time-evolved state with respect to initial and target state (25) for a=1,2,4 and τ=1. Other parameters are set such that ħ=c=1.

In Figure 1, we depict the fidelities for a range of values of *a*. As expected, we see that for a>1, the target state is reached in time τ/a.

## 5. Time-Rescaling Weyl Semimetals: Shortcuts to Adiabatic Pumping

As a second example we discuss time rescaling in the context of adiabatic pumping for Weyl semimetals. To this end, we need to apply the above developed framework to Floquet theory, first.

### 5.1. Floquet Theory and Time-Rescaling

The solution of periodically driven many-body Hamiltonians can be expanded in the so-called Floquet states [56]. To this end, consider a general Hamiltonian [57],

(26) $$H\left(t\right)=\sum_{k}H_{nm}\left(k,t\right)a_{n,k}a_{m,k}^{†}+h.c.$$

where ***k*** is the wave vector in the first Brilloin zone. Here, *a* and $a^{†}$ are the fermionic annihilation and creation operators, and *n* and *m* are indices associated with the system’s degrees of freedom. If this Hamiltonian is periodic in time, H(t)=H(t+T), the single particle wave function can be written as [56,58],

(27) $$\psi \left(k,t\right)=\exp\left\{-\frac{i}{\hbar }\;E\left(k\right)t\right\}\;\phi \left(k,t\right)$$

where ϕ(k,t)=ϕ(k,t+T) is the Floquet mode with periodicity *T*. The quasienergies E(k) are eigenvalues of the Floquet operator equation, and they satisfy,

(28) $$(H\left(k,t\right)-i\hbar \;\partial_{t})\;\phi \left(k,t\right)=E\left(k\right)\;\phi \left(k,t\right).$$

Hence, the E(k) exhibit the corresponding Floquet band structure, if the E(k) are also periodic in momentum space. Interestingly, for fermionic systems, Floquet theories predict a variety of topological states of matter [57], which we will be exploiting in the following.

The major advantage of the Floquet ansatz (27) is that the time evolution operator can be factorized. We have [59],

(29) $$U_{F}\left(nT,0\right)=T_{>}\;\prod_{k=1}^{n}\exp\left\{-\frac{i}{\hbar }\int_{0}^{T}dt\;H\left(t\right)\right\}$$

and $|\psi (nT)\rangle =U_{F}\left(nT,0\right)\;|\psi (0)\rangle$. Thus, it is not difficult to realize that time rescaling can be used to shorten the periodicity of the solutions, and in particular, we can write

(30) $$U_{F}\left(nT,0\right)=T_{>}\prod_{k=1}^{n}\exp\left\{-\frac{i}{\hbar }\int_{f^{-1}\left(0\right)}^{f^{-1}\left(T\right)}ds\;\dot{f}\left(s\right)H\left(f\left(s\right)\right)\right\}$$

where, as above, *s* is the re-scaled time variable and $\dot{f}\left(s\right)$ is the re-scaling function.

If we again consider time acceleration, i.e., $\left|f^{-1}\left(T\right)-f^{-1}\left(0\right)\right|=T/a$ for a>1, then the periodicity of the Floquet states is shorted by a factor of 1/a. In the following, we will exploit this observation for adiabatic evolution between Floquet points, or in other words, will we use time rescaling as a shortcut to adiabatic pumping.

In this context, it is also interesting to note that typically, a periodically driven Hamiltonian does not have to be periodic in momentum space. However, it is still possible to introduce the Floquet operator in terms of a periodic momentum transport variable [60]. Experimentally, this transport variable can be manifested by phase-shifting two optical-lattice potentials in a ratchet accelerator (RA) model [60], and its infinitesimal evolution corresponds to tracing out the entire Floquet band topology, by moving between different eigenstates of the Floquet operator.

### 5.2. Shortcut to Adiabatic Creation of Weyl Points

Only rather recently, it has been shown that Floquet points can exhibit linear dispersion relations. For instance, Refs. [61,62] found topological phases that correspond to Weyl semimetals with three-dimensional Dirac “cones”. This was achieved, by considering a modified, extended kicked Harper model and the careful tuning of “hopping” and “kicking” strengths.

We will now briefly outline how time-rescaling can be applied to create these Weyl points in Floquet theory in shorter time. To this end, we consider the off-diagonal kicked Harper model, whose Hamiltonian reads [62]

(31) $$\begin{array}{cc} H(t) & =\sum_{n=1}^{N-1}\left\{\left[J+{\left(-1\right)}^{n}\lambda \cos\left(\phi_{y}\right)\right]|n+1\rangle \langle n|+h.c.\right\}\\ \; & \;+\sum_{n=1}^{N-1}\sum_{j}{\left(-1\right)}^{n}\left[V_{1}+V_{2}\cos\left(\Omega t\right)\right]\cos\left(\phi_{z}\right)|n\rangle \langle n| \end{array}$$

where *n* is the lattice site index, *J* and λ are parameters controlling the hopping strength, *N* is the total number of lattice sites. Furthermore, $V_{1}$ is the onsite potential, $V_{2}$ represents the coupling with the harmonic driving field, and Ω=2π/T. As before, *T* is the period of driving, and H(t)=H(t+T). In this model [62], $\phi_{y}$ and $\phi_{z}$ are quasimomenta, that can take any value in (−π,π]. Therefore, we can simplify the analysis again to (1 + 1) dimensions. It is also interesting to note, that Weyl chirality can be manifested by observing the time evolution of the mean position, $\langle \Delta x\rangle$. This is facilitated by adiabatic transport in momentum space [60], or rather by the adiabatic variation of $\phi_{y}$ and $\phi_{z}$. Thus, we focus now on applying time rescaling for adiabatic transport to observe the Weyl chirality in $\langle \Delta x\rangle$.

For a single mode *k*, we have [62]

(32) $$H_{k}\left(t\right)=2J\cos\left(k\right)\sigma_{x}+2\lambda \sin\left(k\right)\cos\left(\phi_{y}\right)\sigma_{y}+\left[V_{1}+V_{2}\cos\left(\Omega t\right)\right]\cos\left(\phi_{z}\right)\sigma_{z}\;.$$

It can be shown that the corresponding quasienergy spectrum exhibits band touching points with linear dispersion relation [61,62].

For the time-dependent variation of $\phi_{y}\left(t\right)$ and $\phi_{z}\left(t\right)$, Equation (32) can be written as [62]

(33) $$\begin{array}{cc} \; & {\hat{H}}_{k}\left(t\right)=\left[2J\cos\left(k\right)\cos\left(2\alpha \right)+2\lambda \sin\left(k\right)\cos(\phi_{y}\left(t\right))\sin\left(2\alpha \right)\right]\;\sigma_{x}\\ \; & \;+\left[-2J\cos\left(k\right)\cos\left(2\alpha \right)+2\lambda \sin\left(k\right)\cos(\phi_{y}\left(t\right))\sin\left(2\alpha \right)\right]\;\sigma_{y}+V_{1}\cos\left(\phi_{z}\left(t\right)\right)\;\sigma_{z} \end{array}$$

where $\alpha =V_{2}\cos\left(\phi_{z}\left(t\right)\right)\sin\left(\Omega t\right)/\hbar \Omega$. The corresponding time-rescaled Hamiltonian becomes, ${\tilde{H}}_{k}\left(t\right)\equiv \dot{f}\left(t\right){\hat{H}}_{k}\left(t\right)$, and we now need to verify that ${\tilde{H}}_{k}\left(t\right)$ also exhibits the desired Weyl chirality.

In close proximity of the band-touching bounds [62], we can write

(34) $${\tilde{H}}_{k}\left(t\right)≃\dot{f}\left(t\right)\left\{H_{\mathrm{pert}}+\left[\ell \pi -V_{1}k_{z}\sin\left(\phi_{l}\right)\right]\;\sigma_{z}\right\}$$

where we introduced [62]

(35) $$\begin{array}{cc} H_{\mathrm{pert}} & =\left\{-2Jk_{x}\;\cos\left[\ell c\;\sin\left(\Omega f\left(t\right)\right)\right]-2\lambda k_{y}\;\sin\left[\ell c\;\sin\left(\Omega f\left(t\right)\right)\right]\right\}\;\sigma_{x}\\ \; & \;+\left\{2Jk_{x}\;\sin\left[\ell c\;\sin\left(\Omega f\left(t\right)\right)\right]-2\lambda k_{y}\;\cos\left[\ell c\;\sin\left(\Omega f\left(t\right)\right)\right]\right\}\;\sigma_{y}\;. \end{array}$$

Moreover, we have $k_{x}=k-\pi /2$, $k_{y}=\phi_{y}-\pi /2$, $k_{z}=\phi_{z}-\phi_{l}$, $c=V_{2}/V_{1}$ and $\phi_{l}=\cos^{-1}\left(\ell \pi /V_{1}\right)$. Finally, *ℓ* denotes the quantum number of the quasienergy. Adiabatic momentum transport is then achieved by parametrizing $\phi_{y}$ and $\phi_{z}$ according to $\phi_{y}=\phi_{y,0}+r\cos\left(\theta \left(t\right)\right)$ and $\phi_{z}=\phi_{z,0}+r\sin\left(\theta \left(t\right)\right)$ and evolving θ(t) over a time period $T_{0}\gg T$ [62]. Thus, it is worth emphasizing that the relevant Floquet operator quantifies this periodicity, $T_{0}$, in momentum space, and not the time period of the driving field *T*.

Solving for the time evolution operator exactly is hardly feasible. However, the Floquet operator (29) can be obtained from time-dependent perturbation theory. It can be shown [62] that we have for the first period

(36) $$U_{F}\left(T_{0},0\right)≃I+i\left(2Jk_{x}\;\sigma_{x}+2\lambda k_{y}\;\sigma_{y}\right)J\left(\ell c\right)$$

where J is the Bessel function of the first kind. It is then a simple exercise to show that we obtain for the time-rescaled Floquet operator

(37) $${\tilde{U}}_{F}\left(T_{0}/a,0\right)≃I+i\left(2Jk_{x}\;\sigma_{x}+2\lambda k_{y}\;\sigma_{y}\right)J\left(\ell c\right)$$

which is identical to the Floquet operator (36). However, due to time rescaling, ${\tilde{U}}_{F}$ has a periodicity of $T_{0}/a$, which for a>1, described sped-up dynamics. Since the time-rescaled Floquet operator is identical to the original $U_{F}\left(T_{0},0\right)$, all further steps of the analysis in Ref. [62] remain true, yet the Weyl chirality is obtained with periodicity $T_{0}/a$.

Finally, we briefly remark on the complexity of the time-dependence in the time-rescaled dynamics. As before, in ${\tilde{H}}_{k}\left(t\right)$, all original parameters are multiplied by $\dot{f}\left(t\right)$. In particular, this requires both kicking and hopping strengths to be varied with time. However, it is not hard to see that, in complete analogy to the above in Section 3, the time dependence can be absorbed into potentials. This can be facilitated again with, e.g., the simplest unitary transformation K(t) (13).

## 6. Concluding Remarks

Controlling quantum systems is a ubiquitous goal in the development of quantum technologies. To this end, shortcuts to adiabaticity provide a powerful tool kit to steer quantum system towards desired target states. However, most techniques are rather complicated to be implemented in realistic scenarios, since most of them require exquisite knowledge about the eigenspectrum of the driven Hamiltonians.

Time scaling relies on the simple idea that, rather than controlling single states, faster dynamics can be achieved by simply transforming the dynamics to a new time-frame. To utilize time rescaling as a shortcut to adiabaticty, two criteria need to me met: (i) the original dynamics is adiabatic, and (ii) the resulting Hamiltonian has to be realizable and physical.

Using time rescaling as a shortcut was originally proposed only for Schrödinger dynamics. The natural question arose, whether also relativistic dynamics can be treated in this framework. To answer this question, we have analyzed Dirac dynamics in first quantization. In the second quantization, one has to be concerned with pair production and radiation, and hence a quantum field theoretic description becomes inevitable.

In the present paper, we have shown that time-rescaling can be directly applied to Dirac dynamics, and that the aforementioned criteria are met by at least two experimentally relevant scenarios, namely laser ion traps and the adiabatic creation of Weyl points. Thus, we remain optimistic that time-rescaling will, indeed, find applications in experimental settings.

## Appendix A. Shortcuts to Adiabaticity from Time Rescaling in Scale Invariant Problems

This appendix is dedicated to the question of whether time-rescaling is related to, or even equivalent to, other techniques for shortcuts to adiabaticity. To this end, we analyze the method for scale-invariant problems, in both classical and quantum dynamics.

### Appendix A.1. Classical Hamiltonian Dynamics

We start by considering a classical system that evolves under Hamiltonian, scale-invariant dynamics. For such problems, it has been shown that the counterdiabatic field facilitating transitionless quantum driving takes a particularly simple form [13].

The corresponding Hamiltonian is given by

(A1) $$H\left(x,p,t\right)=\frac{p^{2}}{2m}+\frac{1}{\gamma^{2}}V\left(\frac{x}{\gamma }\right)$$

where *m* is the mass of the particle, γ = γ(t) and *V* are the scale-invariant driving potentials obeying V(x,t)=V(x/γ). Note that time rescaling can be applied directly to classical dynamics, by replacing the commutator in the Heisenberg equation of motion with the Poisson bracket.

The corresponding time-rescaled Hamiltonian becomes

(A2) $$\tilde{H}\left(x,p,t\right)=\dot{f}\left(t\right)\;\frac{p^{2}}{2m}+\frac{\dot{f}\left(t\right)}{\gamma {\left(f\left(t\right)\right)}^{2}}\;V\left(\frac{x}{\gamma (f(t))}\right)$$

which has the same technical problem that we discussed in the main text, namely $\tilde{H}\left(x,p,t\right)$ contains an effectively time-dependent mass. Scaling time is actually a well-studied problem in Hamiltonian dynamics, and it can be expressed in terms of an infinitesimal canonical transformation [63].

Therefore, we now consider a generating function of the second kind [64]

(A3) $$F\left(x,\bar{p},t\right)=h_{1}\left(t\right)\;x\bar{p}+h_{2}\left(t\right)\;x^{2}$$

where $h_{1}$ and $h_{2}$ are two arbitrary functions of time. Accordingly, we have

(A4) $$p=\frac{\partial F}{\partial x}=h_{1}\;\bar{p}+2h_{2}\;x\;\mathrm{and}\;\bar{x}=\frac{\partial F}{\partial \bar{p}}=h_{1}\;x\;.$$

Noting $H\left(\bar{x},\bar{p},t\right)=\tilde{H}\left(\bar{x},\bar{p},t\right)+\partial F/\partial t$, it is then straightforward to show that

(A5) $$\begin{array}{c} H\left(\bar{x},\bar{p},t\right)=\frac{\dot{f}h_{1}^{2}}{2m}\;{\bar{p}}^{2}+\left(\frac{4h_{2}^{2}\dot{f}}{2mh_{1}^{2}}+\frac{\dot{h_{2}}}{h_{1}^{2}}\right)\;{\bar{x}}^{2}+\left(\frac{4h_{2}\dot{f}}{2m}+\frac{\dot{h_{1}}}{h_{1}}\right)\;\bar{p}\bar{x}+\frac{h_{1}^{2}\;f}{{\bar{\gamma }}^{2}}\;V\left(\frac{\bar{x}}{\bar{\gamma }}\right) \end{array}$$

where $\bar{\gamma }=h_{1}\gamma$ and we suppressed the time-dependence of the parameters to avoid clutter.

Choosing the arbitrary functions $h_{1}$ and $h_{2}$ as

(A6) $$h_{1}=1/\sqrt{\dot{f}}\;\mathrm{and}\;h_{2}=m\ddot{f}/4{\dot{f}}^{2}$$

the non-local term disappears and the time-dependence of the kinetic terms is remedied. Hence, we can write

(A7) $$H\left(\bar{x},\bar{p},\tau \right)=\frac{{\bar{p}}^{2}}{2m}+\frac{\dot{f}}{\gamma^{2}}\;V\left(\frac{\bar{x}\sqrt{\dot{f}}}{\gamma }\right)+\kappa {\bar{x}}^{2}$$

with $\kappa =m{\ddot{f}}^{2}/8{\dot{f}}^{2}+m\left(\dot{f}\ddot{f}-2{\ddot{f}}^{3}\right)/4\dot{f}$.

In conclusion, we have shown that time-rescaling in scale-invariant problems is equivalent to including an auxiliary field, which is simply given by a harmonic term. Since it also has been shown that the counterdiabatic field in transitionless quantum driving can be reduced to a harmonic term [13], we have that time-rescaling can indeed facilitate transitionless quantum driving.

### Appendix A.2. Scale-Invariant Schrödinger Dynamics

The analogous problem can also be solved for Schrödinger dynamics. In this case, we write the quantum generating function as

(A8) $$F\left(x,p,t\right)=\exp\left\{\frac{i}{\hbar }\;\alpha \left(t\right)x^{2}\right\}\exp\left\{\frac{i}{\hbar }\;\beta \left(t\right)\left\{x,p\right\}\right\}$$

for two arbitrary functions α(t) and β(t), and {x,p}=xp+px. Now choosing,

(A9) $$\beta \left(t\right)=\log\left(\dot{f}\left(t\right)\right)\;\mathrm{and}\;\alpha \left(t\right)=\frac{\ddot{f}\left(t\right)}{\dot{f}\left(t\right)}\;,$$

we obtain for $\tilde{H}\left(t\right)=\dot{f}\left(t\right)\;H\left(t\right)$

(A10) $$H\left(x,p,t\right)=\frac{p^{2}}{2m}+\frac{\dot{f}}{\gamma^{2}}\;V\left(\frac{x}{\dot{f}\gamma }\right)+\kappa_{q}\;x^{2}\;,$$

and $\kappa_{q}=\dddot{f}/\dot{f}-{\ddot{f}}^{2}/{\dot{f}}^{2}-{\ddot{f}}^{2}/{\dot{f}}^{3}$. In complete analogy to the classical case, time-rescaling in the original frame is facilitated by an auxiliary harmonic term.
